# *Plasmodium berghei* circumsporozoite protein encapsulated in oligomannose-coated liposomes confers protection against sporozoite infection in mice

**DOI:** 10.1186/1475-2875-13-426

**Published:** 2014-11-05

**Authors:** Mohamad Alaa Terkawi, Yasuhiro Kuroda, Shinya Fukumoto, Sachi Tanaka, Naoya Kojima, Yoshifumi Nishikawa

**Affiliations:** National Research Center for Protozoan Diseases, Obihiro University of Agriculture and Veterinary Medicine, Inada-cho, Obihiro, Hokkaido 080-8555 Japan; Department of Applied Biochemistry, Tokai University, Kita-kaname, Hiratsuka, Kanagawa 259-1292 Japan

**Keywords:** Vaccine, *Plasmodium berghei*, Oligomannose-coated liposome

## Abstract

**Background:**

The design and development of an effective malaria vaccine against the pre-erythrocytic and erythrocytic-stages of infection present a great challenge.

**Methods:**

In the present study, protective efficacy of oligomannose-coated liposome (OML)-entrapped merozoite and sporozoite antigens against *Plasmodium berghei* challenge infection in BALB/c mice was evaluated.

**Results:**

Subcutaneous immunization with truncated merozoite surface protein 1 entrapped with OML (OML-PbMSP1) prolonged survival, but failed to protect the mice from erythrocytic-stage infection, despite the antigen-specific antibody responses induced by the immunization regimen. In contrast, immunization with circumsporozoite protein entrapped with OML (OML-PbCSP) elicited antigen-specific humoral and cellular responses, which correlated with substantial protection against sporozoite challenge infections.

**Conclusions:**

The current results represent the use of an oligomannose-coated liposome-based vaccine against pre-erythrocytic and erythrocytic stages malaria infection. This approach may offer a new vaccination strategy against malaria infection.

## Background

Malaria remains the most important parasitic disease of humans, affecting 40% of the world’s population and causing over 600,000 deaths annually [1]. Vaccination to prevent the infection is believed to be the most realistic approach for reducing malaria morbidity and mortality. Over the last decades, the gold standard for malaria vaccine development has been immunization with radiation-attenuated sporozoites [1]. Recent study has shown that genetic attenuated sporozoites by gene deletion may offer better strategy for development of malaria vaccine [2]. Although this vaccine strategy offers sterile protection against infection, several problems exist that have hampered its use including the cost, dose standardization, production, sporozoite irradiation, and immunization-related issues [3, 4].

The most advanced malaria vaccine to date, RTS,S, comprises a portion of the *Plasmodium falciparum* circumsporozoite protein (CSP) central repeat (NANP) and C-terminal region, which contains T cell epitopes fused to the hepatitis B virus surface antigen. Since early 1996, several adjuvant formulations of this vaccine have been tested against live sporozoite challenge in volunteers, with the highest protective efficacies (30–50%) observed with an adjuvant containing monophosphoryl lipid A and QS21 (a derivative of Quill A) [5, 6]. In addition, the prime-boost regimen for the modified vaccinia virus Ankara (MVA) and the new fowlpox FP9 strain, both of which encode the *Plasmodium falciparum* thrombospondin-related adhesion protein (TRAP), offered some degree of protection in African volunteers [7]. Asexual erythrocytic-stage vaccines, tested in clinical trials, also afford some degree of protection [8]. Two asexual-stage proteins, merozoite surface protein 1 (PfMSP1) and apical membrane antigen 1, are the most extensively studied candidates for the development of erythrocytic-stage vaccines [8, 9]. Remarkably, all candidates for subunit vaccines against malaria target the parasite invasion process into the host cells. Despite the promising levels of protection induced by these vaccines, none appear potent enough to completely prevent infection in the majority of recipients. Therefore, further vaccine development research is required to obtain the ultimate goal of complete prevention of malarial infection.

Accumulating evidence shows that protective immunity against liver-stage malaria parasites requires Th1-type immune responses; these responses orchestrate optimal CD8^+^ T cell-mediated cytotoxicity responses, CD8^+^ T cells act as the principal effector cells for elimination of infected hepatocytes, and induction of neutralizing antibodies for trapping extracellular sporozoites [10, 11]. Elimination of erythrocytic-stage parasites is dependent on CD4^+^T cells; they release cytokines that activate other effector cells to clear parasite-infected red blood cells (pRBCs) and maintain protective antibody production. The mechanisms by which antibodies are effective include blockading the invasion of free merozoites into RBCs and cytophilic antibody-dependent cellular killing [12]. Therefore, ideal vaccines for pre-erythrocytic and erythrocytic-stage malaria should induce a protracted Th1 memory response coupled with a sufficiently robust parasite-neutralizing antibody response [12, 13]. Such responses are achievable using appropriate immunization regimens in conjunction with novel adjuvants and vaccine delivery vehicles [14].

Oligomannose-coated liposomes (OML) are a novel adjuvant capable of inducing Th1 immune responses and cytotoxic T lymphocytes (CTLs) specific for the encased antigen. OMLs are taken up preferentially by phagocytic cells, such as dendritic cells and macrophages, through mannose-binding lectin receptors and complement receptor type 3, which leads to antigen-presenting cell (APC) maturation, expression of co-stimulatory major histocompatibility complex (MHC) class I and II molecules, and migration of APCs into lymphoid tissues from peripheral tissues. Consequently, APCs introduce the encapsulated protein to CD4^+^ and CD8^+^ T cells, which generate encased-antigen-specific Th1 cells and CTL responses in the host [15, 16]. Indeed, the protective effects of OML-based vaccines have been reported for a variety of protozoan infections, including those caused by *Neospora caninum*, *Leishmania major* and *Toxoplasma gondii* [17–21]. In the present study, the protective effects of immunization with OML-entrapped C-terminal-merozoite surface protein 1 (OML-PbMSP1) and truncated circumsporozoite protein (OML-PbCSP) against *Plasmodium berghei* merozoite and sporozoite challenge in BALB/c mice were investigated. The truncated regions of each antigen used for OML encapsulation were designed based on the earlier vaccination trials with protective effects or their role in host cells invasion [22]. Moreover, truncated region of PbCSP was designed based on the target region of RTS,S vaccine containing CD8 epitopes and repeat region of CSP [5, 23]. The present study revealed that the OML-PbCSP immunization regimen conferred a significant degree of protection against the pre-erythrocytic stage of *P. berghei*.

## Methods

### Ethics statement

This study was performed in strict accordance with the recommendations in the Guide for the Care and Use of Laboratory Animals of Ministry of Education, Culture, Sports, Science and Technology, Japan. The protocol was approved by the Committee on the Ethics of Animal Experiments of the Obihiro University of Agriculture and Veterinary Medicine (Permit number 24–17, 25–66). All surgery was performed under isoflurane anesthesia, and all efforts were made to minimize animal suffering.

### Mice

Seven-week-old BALB/c mice purchased from Clea (Tokyo, Japan) were housed under specific-pathogen-free conditions in the animal facility of the National Research Center for Protozoan Diseases at Obihiro University of Agriculture and Veterinary Medicine, Obihiro, Japan. All mice used in the present study were treated under the guiding principles for the care and use of research animals promulgated by Obihiro University of Agriculture and Veterinary Medicine.

### Parasites

*Plasmodium berghei* (ANKA strain) was obtained from the Department of Molecular Parasitology, Ehime University School of Medicine, Japan and maintained by mosquito transmission in *Anopheles stephensi* interspersed by a maximum of two serial passages in BALB/c mice. The pRBCs were recovered from frozen pRBC stock by intraperitoneal (i.p.) passage inoculations in mice. Sporozoites were obtained by dissection of salivary glands from *P. berghei*-infected female mosquitos 21 days after taking blood meals from *P. berghei* infected mice.

### Recombinant proteins and liposome preparation

**Figure 1:**
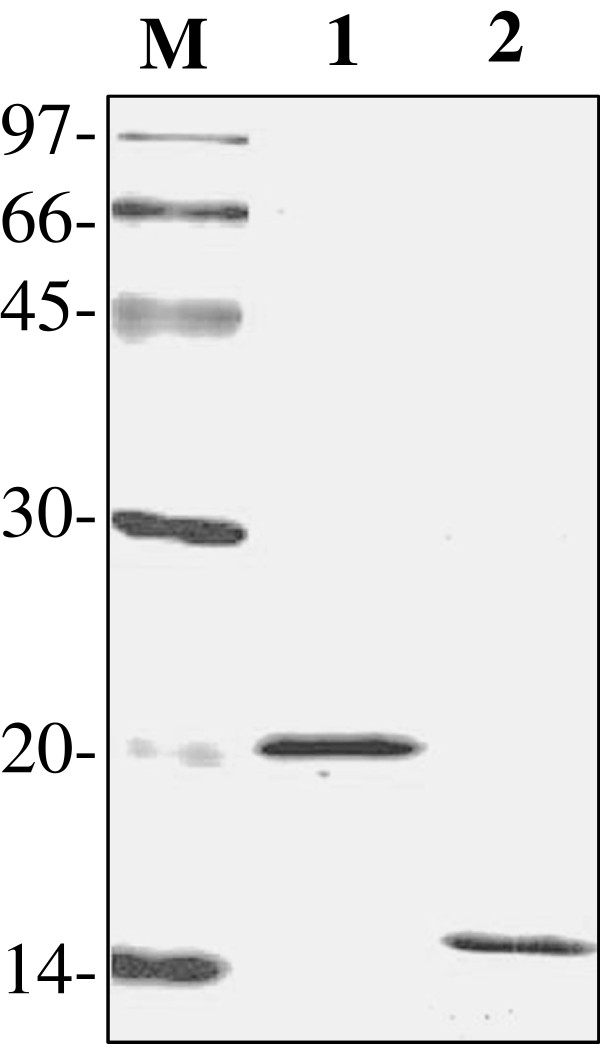
**Expression and purification of recombinant proteins.** 15% sodium dodecyl sulphate polyacrylamide electrophoresis gel (SDS-PAGE) for recombinant proteins stained with Coomassie blue. Lanes: M, molecular mass marker; lane 1, PbMSP1; lane 2, PbCSP.

Recombinant proteins comprising truncated regions of PbMSP1 (Leu_1609_-Ser_1768_, GenBank accession number: AAC28871) and PbCSP (Asn_201_-Asn_347_, GenBank accession number: P23093.1) were produced in *Escherichia coli* as glutathione S-transferase (GST) fusion proteins. Briefly, the coding regions of the targeted genes were amplified from *P. berghei* ANKA genomic DNA with specific primer sets designed from the GenBank sequences: these were 5’-AC **GGA TCC** AGT ATT ACC ACC GAG CAG AA-3’ , which includes a *Bam*HI restriction enzyme site (boldface), and 5’-AG **CTC GAG** TTA GCT GGA AGA GCT ACA GAA-3’ , which includes a *Xho*I restriction enzyme site (boldface) for PbMSP; and 5’-AA **GGA TCC** CAG CCA CAA CCA CAG CCA GGT-3’ , which includes a *Bam*HI restriction enzyme site (boldface), and 5’-GG **CTC GAG** TTA TGA ACA TTT ATC CAT TTT-3’ , which includes a *Xho*I site (boldface) for PbCSP. PCR products were digested with the appropriate restriction enzymes and then ligated into a pGEX-4 T1 (GE Healthcare, Buckinghamshire, UK) expression vector, which had been digested with the same set of restriction enzymes. The nucleotide sequences of the PbMSP1- and PbCSP-positive plasmid inserts were determined using an ABI 3100 DNA sequencer (Applied Biosystems, Foster City, CA, USA). Recombinant proteins were expressed in large-scale DH5α strain *E. coli* cultures (Takara Bio Inc., Osaka, Japan) and purified by Glutathione-Sepharose 4B beads (GE Healthcare). Purified recombinant proteins were treated with thrombin protease (GE Healthcare) to digest the GST tag and then subjected to endotoxin removal using the membrane filter, Acrodisc® Unite (Pall Life Sciences, Ann Arbor, MI, USA). The purity and quantity of the recombinant proteins were tested by sodium dodecyl sulphate polyacrylamide gel electrophoresis (SDS-PAGE) stained with Coomassie Brilliant Blue R250 (MP Biomedicals Inc., Illkirch-Graffenstaden, France) and by a BCA protein assay kit (Thermo Fisher Scientific, Inc. Rockford, IL, USA). Single bands (20-kDa and 14-kDa) corresponding to each recombinant protein (PbMSP-1 and PbCSP, respectively) were observed with SDS-PAGE (Figure 1). Thereafter, OMLs were prepared for recombinant protein entrapment as described previously [17–21], and the amount of entrapped antigen was measured using a modified Lowry protein assay reagent (Pierce, Rockford, IL, USA).

### Immunization and challenge infections

Seven-week-old female BALB/c mice were immunized subcutaneously (s.c.) with 3 μg of OML-PbMSP1 or OML-PbCSP. In parallel, the other groups of mice were immunized with 3 μg of the corresponding naked protein, or OML alone. Two boosts with the same dose of protein were administered s.c. at 14-day intervals. In separate experiments, modified immunization regimens that included boosting once before infection and immunization with 1 μg of OML-PbCSP were performed. In all experiments, each mouse received a 100 μl immunization, adjusted by adding phosphate-buffered saline (PBS). Serum samples (15 μl) were serially obtained from the tail vein of each mouse at 0, 14, 28 and 42 days post-1^st^ immunization. The specific reactivity of immune sera was confirmed by using indirect fluorescence assay with pRBCs or purified sporozoites. Thereafter, mice immunized with PbMSP1 were infected with an intraperitoneal (i.p.) inoculation of 1 × 10^5^ fresh pRBCs, while mice immunized with PbCSP were infected s.c. (into the loose skin over the neck and shoulders) with a live inoculum of 2 × 10^3^ sporozoites/mouse 14 days after the last boost. Parasitemia and survival rates were monitored over a 30-day period.

### Measurement of antigen-specific antibodies

An enzyme-linked immunoabsorbent assay (ELISA) was performed to examine the immunogenicity of the immunization regimen as described previously [18, 20, 21]. Briefly, 50 μl of purified antigen (at a final concentration of 0.1 μM) was coated onto a 96-well microtiter plate (Nunc, Roskilde, Denmark) and incubated overnight at 4°C with a carbonate-bicarbonate buffer (pH 9.6). Plates were blocked with 100 μl of PBS-3% skimmed milk (3% SM-PBS) per well and then incubated at 37°C for 1 h with 50 μl of each serum sample diluted 1:100 or serially diluted from 1:100 to 1:51,200 in 3% SM-PBS. After washing six times with PBS containing 0.05% Tween 20, the plates were incubated at 37°C for 1 h with horseradish peroxidase-conjugated anti-mouse IgG, IgG1 or IgG2a (Bethyl Laboratories, Montgomery, TX, USA) diluted in 3% SM-PBS to 1:4,000. Thereafter, the plates were washed with 100 μl substrate solution (0.1 M citric acid, 0.2 M sodium phosphate, 0.003% H_2_O_2_), and 0.3 mg/ml 2,2’-azide-bis [3-ethylbenzthiazoline-6-sulfonic acid] (Sigma St. Louis, MO, USA) was added to each well. The mean optical density (OD) of the wells was determined by measurement at a wavelength of 415 nm using an MTP-500 microplate reader (Corona Electric, Tokyo, Japan). Endpoint titers were expressed as the reciprocal of the highest sample dilution for which the OD was equal or greater than the cut-off values calculated based on the average ODs of the pre-immune mouse sera plus three standard deviations.

### *In vitro*splenocyte stimulation assays

Spleens were harvested 14 days after the last booster, and a single-cell suspension of splenocytes was plated into 96-well microplates at 5 × 10^5^/200 μl/well in RPMI 1640 medium (Sigma) supplemented with 5% foetal bovine serum [18, 21]. Cultures were stimulated by adding 10 or 50 μg/ml of purified PbCSP, or 0.5 μg/ml of concanavalin A. After incubation for 48 h at 37°C, the culture supernatants were collected and assayed for IFN-γ, IL-4 and IL-10 production using commercial ELISA kits (Pierce Biotechnology, Rockford, IL, USA), according to the manufacturer’s instructions.

### Statistical analysis

The significant differences among the means of all variables were examined by a one-way analysis of variance followed by Tukey’s multiple comparison test (GraphPad Prism 5, GraphPad Software Inc., San Diego, CA, USA). Results were considered to be statistically significant when p was <0.05.

## Results

### Immunization with OML-PbMSP1 prolonged survival but fails to protect mice from erythrocytic-stage challenge infection with *Plasmodium berghei*

**Figure 2:**
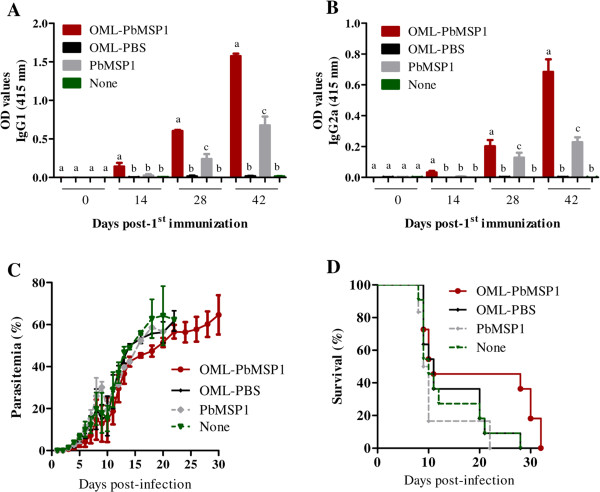
**Efficacy of immunization with OML-PbMSP1.** ELISA detection of antigen-specific IgG1 **(A)** and IgG2a **(B)** in mice immunized with recombinant PbMSP1 over the course of immunization. The mean optical density (OD) was determined at a wavelength of 415 nm. Each bar represents the mean ± SD for used mice per group and results are representative of two independent experiments. Different superscript letters indicate statistically significant differences (*P* <0.05) among groups at each time-point as determined by a one-way analysis of variance followed by Tukey’s multiple comparison test. Parasitemias **(C)** and survival rates **(D)** after challenge infection with pRBCs. Each bar represents the mean ± SD for 11 mice per group (only 6 mice for PbMSP1) and results are from two pooled independent experiments. Mice were either immunized by OML-PbMSP1 (OML-PbMSP1), OML alone (OML-PBS), or naked PbMSP1 (PbMSP1), or not immunized (None).

To evaluate the immunogenicity of the OML-PbMSP1 immunization, sera were serially sampled prior to and after each immunization, and the antibody responses were examined by ELISA using PbMSP1. Of note, OML-PbMSP1 induced highly specific antibody responses in the mice consisting of IgG1 and IgG2a isotypes (Figure 2A,B). Indeed, the PbMSP1-specific antibodies induced by immunization with OML-PbMSP1 were significantly greater than those induced by naked antigen over the course of the immunizations, and it was found that the titers increased at least 10-fold after the third immunization (Table 1 and Figure 2A,B). No PbMSP1-specific antibody responses were detected in mice that received OML or no immunization. Thereafter, to evaluate the protective efficacy of OML-PbMSP1 against erythrocytic-stage infection, mice were infected with pRBCs and their parasitemia and survival rates were monitored over a 30-day period. Mice showed a patent parasitemia by day 3 post-infection and succumbed to malaria infection within one month (Figure 2C,D). Notably, the OML-PbMSP1-immunized mice had a delay in their onset of parasitemia (36.4%, 4/11 mice) and prolonged survival as compared with the other groups of mice (Table 1).

**Table 1:** **Effects of OML-PbMSP1 immunization against** ***P. berghei*** **merozoite challenge**

Immunization	Antibody titration*		Infected/total [l ^st^+2 ^nd^trials]	Survival*	Protection ^†^
	IgGl	IgG2a	(Number)	(Day)	(%)
OML-PbMSP1	11636 ± 5593^††^	2182 ± 1604^††^	11/11 [(5/5) + (6/6)]	19.1 ± 10.9	0
PbMSP1	1333 ± 413	283 ± 132	6/6	14.3 ± 6.7	0
OML-PBS	0	0	11/11 [(5/5) + (6/6)]	11.3 ± 5.3	0
None	0	0	11/11 [(5/5) + (6/6)]	11.8 ± 5.2	0

*Antigen-specific 1gG1 and IgG2a detected by ELISA with recombinant PbMSP1 at 14 days after the last boost. Each value represents the mean titer of antibody ± SD per mouse group.

*Each value represents the average survival day ± SD per mouse group.

^†^Mice were scored as protected when parasitemia was not observed over a 30-day period.

^††^Indicates statistically significant differences between the antibody titers of OML-PbMSP1-immunized mice and those of the corresponding mice immunized with naked antigen.

Results represent pooled data of two independent experiments.

### Immunization with OML-PbCSP confers substantial protection against liver-stage *Plasmodium berghei*infection

**Figure 3:**
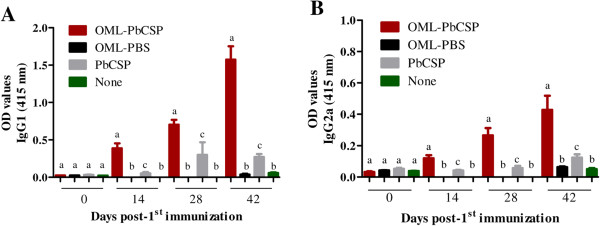
**Humoral responses in mice to immunization with OML-PbCSP.** ELISA detection of antigen-specific IgG1 **(A)** and IgG2a **(B)** in mice immunized with recombinant PbCSP. The mean optical density (OD) was determined at a wavelength of 415 nm. Each bar represents the mean ± SD for used mice per group and results are representative of two independent experiments. Different superscript letters indicate statistically significant differences (*P* <0.05) among groups at each time-point as determined by a one-way analysis of variance followed by Tukey’s multiple comparison test. Mice were either immunized by OML-PbCSP (OML-PbCSP), OML alone (OML-PBS), naked PbCSP (PbCSP) or not immunized (None). Different superscript letters indicate statistically significant differences (*P* <0.05) among groups as determined by a one-way analysis of variance followed by Tukey’s multiple comparison test.

The immunization regimen with either OML-PbCSP (3 μg) induced strong and specific antibody responses to the antigen, which consisted of IgG1 and IgG2a isotypes (Figure 3A-B). Antigen-specific IgG1 and IgG2a were significantly greater in mice immunized with OML-PbCSP than in mice immunized with the corresponding naked antigen over the course of the immunizations (Figure 3A-B). No specific-PbCSP antibody responses were observed in mice that received OML or no immunization. Consistently, antibody titration against antigen revealed that the IgG1 and IgG2a titers increased at least 20-fold and 16-fold, respectively, in the OML-based antigen-immunized mice as compared with those that received the corresponding naked antigen (Table 2). These results showed that the immunization regimen for OML-PbCSP was capable of inducing robust humoral responses consisting of IgG1 and IgG2a isotypes. Next, immunized mice were infected s.c. with a live inoculum of 2 × 10^3^ sporozoites/mouse 14 days after their last boost, after which their parasitemia and survival rates were monitored over a 30-day period. Notably, 54% of the OML-PbCSP-immunized mice showed complete sterile protection, as defined by the absence of patent parasitemia over the study period. In contrast, the OML-immunized control group showed 9.1% protection (Table 2). No protection was observed in the mice that received the naked antigen immunization regimen or no immunization (Table 2). Moreover, comparison of the parasitemia curves revealed that the OML-antigen-immunized mice, which had pRBCs, experienced at least a 1-day delay in the onset of parasitemia as compared with mice immunized with naked antigen or OML alone, or those that were not immunized (Table 2). Moreover, antibody titer of anti-PbCSP-specific IgG2a in protected mice by OML-PbCSP immunization tended to be higher than those in susceptible mice received same immunization (protected; 3520 ± 787, unprotected; 1733 ± 1753, *P* = 0.0504), while there was no significant difference in the antibody titer of the specific IgG1. Furthermore, use of a modified immunization regimen comprising OML-PbCSP with a single boost resulted in a reduced protection rate as noted as 16.7% (Table 2). The immunization regimen of OML-PbCSP containing 1 μg antigen with two boots resulted in 66.7% complete protection in mice. Nonetheless, lower protection correlated with reduced antibody responses was observed in OML-PbCSP-immunized mice with single boost (Table 2). These results show that the immunization regimen with OML-PbCSP conferred a significant degree of protection against pre-erythrocytic stage infection of *P. berghei* in the BALB/c mice.

**Table 2:** **Effects of OML-based antigen immunization against** ***P. berghei*** **sporozoite challenge**

Antigen	Immunization		Antibody titration*		Infected/total [1 ^st^+ 2 ^nd^trials] (Number)	Parasitemia*	Protection ^†^(%)
	Dose	Boost	IgG1	IgG2a			
OML-PbCSP	3 μg	2	10666.7 ± 3304.9^††^	3466.7 ± 1573.1^††^	5/11 [(2/5) + (3/6)]	6.40 ± 0.55	54% (*P* = 0.004)
	1 μg	2	4900.0 ± 2038.6^††^	1200.0 ± 979.8^††^	2/6	8.50 ± 2.10	66.7% (*P* = 0.002)
OML-PbCSP	3 μg	1	1733.3 ± 786.5^††^	600.0 ± 219.1^††^	5/6	6.50 ± 1.00	66.7% (*P* = 0.002)
	1 μg	1	533.3 ± 206.6^††^	266.7 ± 103.3^††^	4/6	6.60 ± 1.34	33.3% (*P* = 0.041)
PbCSP	3 μg	1	433.3 ± 196.6	166.7 ± 51.6	11/11 [(5/5) + (6/6)]	5.83 ± 0.98	0% (N.A.)
OML-PBS		2	0	0	10/11 [(5/5) + (5/6)]	5.50 ± 1.40	5.9% (*P* = 0.413)
None		0	0	0	11/11 [(5/5) + (6/6)]	5.09 ± 1.04	0%

*Antigen-specific IgG1 and IgG2a detected by ELISA with recombinant PbCSP l4 day after the last boost. Each value represents the mean antibody titer ± SD per mouse group.

*Each value represents tie mean day of parasitemia onset ± SD per mouse group.

^††^Indicates a statistically significant difference between the antibody response of the OML-based antigen-immunized group to those of the corresponding group immunized with naked antigen.

^†^Mice were scored as protected when parasitemia was not observed over a 30-day pariod.

^†^Significant difference in protection rate as compared to none and as calculated by chi-test. NA.: Not applicable.

Results represent pooled data of two independent experiments.

**Figure 4:**
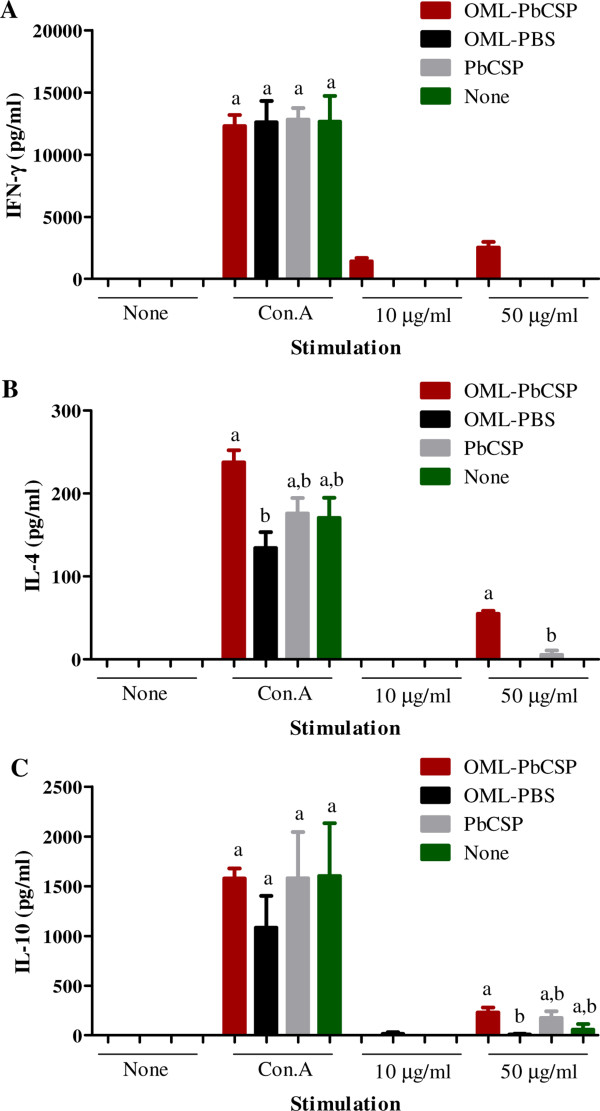
**Cytokine detection in splenocyte cultures.** To detect IFN-γ **(A)**, IL-4 **(B)** and IL-10 **(C)** production by ELISA, splenocyte cultures for each group of mice (n = 4) were prepared and the supernatants were collected after incubation for 48 h at 37°C with the PbCSP antigen (10 or 50 μg/ml), 0.5 μg/ml of concanavalin A (ConA) or without any stimulus. Each bar represents the mean ± SD based on four mice per group. Mice were either immunized by OML-PbCSP (OML-PbCSP), OML alone (OML-PBS), naked PbCSP (PbCSP), or not immunized (None). Different superscript letters indicate statistically significant differences (*P* <0.05) among groups as determined by a one-way analysis of variance followed by Tukey’s multiple comparison test.

Additionally, to gain better insight into the cellular responses induced by the immunization regimen with OML-PbCSP, splenocytes were prepared from immunized mice and stimulated *in vitro* for cytokine detection. Strikingly, splenocytes from the OML-PbCSP-immunized mice released high levels of IFN-γ after stimulation with PbCSP at concentrations of 10 μg/ml or 50 μg/ml (Figure 4A). In contrast, the IFN-γ levels of the splenocytes from OML- or naked PbCSP-immunized mice as well as non-immunized mice after stimulation with the same antigen were below the limits of detection of the kits. Likewise, IL-4 was detected in cultures from the antigen-immunized mice after stimulation with 50 μg/ml PbCSP (Figure 4B). However, detected IL-4 levels in OML-PbCSP-immunized mice were significantly higher than those detected in naked antigen immunized mice (Figure 4B). Levels of IL-10 were not significantly different in all cultures after stimulation with 50 μg/ml PbCSP (Figure 4C). Stimulation with concanavalin A, which acted as a positive control, resulted in high levels of IFN-γ, IL-4 and IL-10 in all of the cultures (Figure 4).

## Discussion

In general, protection against erythrocytic-stage infection is thought to be mediated by CD4^+^ T cells, which orchestrate the activation of effector cells and maintain the production of protective antibodies. Humoral responses have been shown to play an indispensable role in protection against malaria by blocking merozoite invasion and neutralizing pRBCs for macrophage phagocytosis [24, 25]. However, subunit vaccine failure against *P. berghei* erythrocyte-stage parasite challenge has often been observed [26, 27], with only a few trials showing some degree of protection in mouse model [22, 28]. The difficulties in achieving protection against *P. berghei* might be caused by suppression or evasion of the immune system during the infection. In fact, alongside the sequestration of parasites in blood capillaries, erythrocytic-stage *P. berghei* can rapidly suppress MHC class I and class II presentation of malarial antigens by APCs, thereby preventing the development of protective immunity in vaccinated mice [27, 29]. A recent study has shown that vaccination of mice with recombinant *P. berghei* schizont egress antigen-1 (PbSEA-1) significantly reduces parasitemia and delays mortality. The authors have concluded that the protection by PbSEA-1 is due to the function of its specific antibody that decreases the parasite replication by arresting schizont and preventing the egression [30]. In the present study, immunization with OML-PbMSP1 prolonged survival but failed to protect mice against the infection with erythrocytic-stage *P. berghei.* However, immunizations with the OML-PbMSP1 induced the production of anti-PbMSP1 IgG1 and IgG2a, which recognized the parasites. Thus, a possibility for lack of protection in this data may be due to the insufficient antibody titer against the parasite infection.

Sterile protection induced by an effective malaria vaccine should lead to the blockade of exoerythrocytic forms retained within hepatocytes and trapped by Kupffer cells, whereas activated CTLs act as effector cells for the elimination of parasitized hepatocytes in the liver [9, 10]. Moreover, CD4^+^ T cells seem to contribute to protective immunity in the liver through initiating the responses of CD8^+^ T effector cells and mediating the production of neutralizing antibodies [31]. Sporozoite-specific antibodies may neutralize malaria parasites and inhibit their invasion into hepatocytes, thereby allowing macrophages and other polymorphonucleocytes to phagocytize extracellular sporozoites [9, 10]. In addition, a recent study has shown that sporozoite-specific antibodies are protective and provide sterilizing immunity against malaria infection when reaching or exceeding a critical plasma concentration [32]. In the present study, immunization with OML-based sporozoite antigen induced high humoral responses consisting of IgG1 and IgG2a, and the splenocyte cultures from the OML-PbCSP-immunized mice released the production of IFN-γ, IL-4 and IL-10 after stimulation with the PbCSP*.* These results support the concept that OML has adjuvant properties through triggering humoral and cellular responses [15–21]. Strikingly, immunization with OML-PbCSP elicited substantial protection in mice against sporozoite challenge infection. The protection conferred by s.c. immunization with OML-PbCSP may be related to activation of professional APCs by OML; these cells might effectively present PbCSP-derived peptides via MHC class I and class II molecules, which prime specific CD8^+^ and CD4^+^ memory T cells, respectively [15]. Thus, effector memory T cells may migrate into the liver and trigger local CTLs that eliminate parasitized hepatocytes following challenge infection with sporozoites. In parallel, PbCSP-specific antibodies might inhibit sporozoite invasion and mediate the clearance of parasites by antibody-dependent-cellular-cytotoxicity. Indeed, the present data revealed some correlation between the protection and the antibody response to PbCSP as evidenced by elevated antibody titers in protected mice over unprotected received same immunization regimen.

Nonetheless, the protection rates observed in the present data were comparable to those in several other immunization trials using murine models of infection. For instance, immunization with the long synthetic polypeptide, PbCSP_242–310_, coupled with the QS-21 saponin adjuvant resulted in a 60% level of protection [23]. Administration of recombinant adenylate cyclase toxoid of *Bordetella pertussis* containing a PbCSP epitope elicited a robust IFN-γ-producing T cell response also associated with 60% protection in mice in the absence of further adjuvant [33]. Additionally, immunization with the repeat epitope of PbCSP and different oil-based adjuvants elicited 29–100% sterile protection in mice [34]. Genetic vaccination with PbCSP induced 30–90% protection in mice against sporozoite and mosquito challenge infections [35]. Likewise, prime-boost vaccinations of the attenuated recombinant poxviruses MVA and FP9 encoding CSP or thrombospondin-related adhesion protein of *P. berghei*, respectively, were shown to confer substantial protection (20–90%) against liver-stage murine malaria [7, 36].

OMLs are preferentially incorporated into macrophages via ICAM-3 grabbing nonintegrin-related 1 (SIGNR1) [15, 37, 38]. Furthermore, the macrophages and DCs also produce IL-12 in response to the preferential uptake of OMLs, leading to antigen-specific Th1 immunity [39]. Thus, the uptake of antigen-encapsulating OMLs by APCs must be an initial key event in the induction of the antigen-specific Th1 immune response. OMLs could be defined as good adjuvant that enhanced the immunogenicity of antigenic vaccine components. In addition, OMLs are very suitable for use as an adjuvant and vehicle for vaccines because they consist of innocuous materials distributed ubiquitously throughout the body and cause no damage to the skin at the injection site [40]. To develop novel type of malaria vaccine, there is a need to evaluate the enhanced protection of OML-based vaccine by using appropriate adjuvant such as alum, cocktail antigens or synthetic peptides formation.

## Conclusions

Immunization with OML-PbCSP elicited substantial protection against liver-stage malaria infection in BALB/c mice. The current approach has potential to provide a novel antigen-delivery system for developing malaria vaccine.

## Abbreviations

OML: Oligomannose-coated liposome
OML-PbMSP1: *Plasmodium berghei* Merozoite surface protein 1 entrapped with OML
OML-PbCSP: *Plasmodium berghei* Circumsporozoite protein entrapped with OML
pRBCs: Infected red blood cells
APC: Antigen-presenting cell
MHC: Major histocompatibility complex
CTLs: Cytotoxic T lymphocytes
i.p.: Intraperitoneal
s.c.: Subcutaneous
IFN-γ: Interferon gamma
IL-4: Interleukin 4
ELISA: Enzyme-linked immunoabsorbent assay
PBS: Phosphate buffered saline
OD: Optical density
SD: Standard deviation.
